# Obstetric and perinatal outcomes following frozen and fresh embryo transfer in patients with endometrial hyperplasia and carcinoma: a retrospective study in a high-volume reproductive center

**DOI:** 10.1186/s12884-023-05418-7

**Published:** 2023-02-03

**Authors:** Xuan Zong, Yaxing Guo, Hongzhen Li, Rong Li, Jie Qiao

**Affiliations:** 1 Department of Obstetrics and Gynecology, Center for Reproductive Medicine, Peking University Third Hospital, Beijing, 100191 China; 2 National Clinical Research Center for Obstetrics and Gynecology, Peking University Third Hospital), Beijing, 100191 China; 3 Key Laboratory of Assisted Reproduction (Peking University), Ministry of Education, Beijing, 100191 China; 4 Beijing Key Laboratory of Reproductive Endocrinology and Assisted Reproductive Technology (Peking University Third Hospital), Beijing, 100191 China

**Keywords:** Live birth, Maternal outcome, Frozen embryo transfer, Endometrial atypical hyperplasia, Endometrial cancer

## Abstract

**Background:**

There is ongoing debate regarding which embryo transfer procedure can achieve a higher live birth rate. Research has suggested that frozen ET might be beneficial for certain populations, such as hyper-responders. This study aimed to compare outcomes of pregnancies between frozen and fresh embryo transfer cycles in patients with endometrial hyperplasia and carcinoma.

**Methods:**

This retrospective cohort study was conducted at a high-volume reproductive center from January 2010 to January 2022. Patients who were diagnosed with endometrial hyperplasia with atypia and endometrial carcinoma were included. They all underwent in vitro fertilization after conservative treatment. The primary outcome was live birth after frozen and fresh embryo transfer cycles, and secondary outcomes included perinatal complications and other pregnancy outcomes.

**Results:**

Overall, 259 ET cycles (130 fresh and 129 frozen) were included. The rate of live births per embryo transfer cycle of the whole cohort was 20.8% (54/259), and no significant between-group difference was found after adjusting for potential confounding factors (23.8% vs. 17.8%; adjusted OR, 0.47; 95% CI, 0.21-1.06; *p*=0.068). Compared to fresh embryo transfer group, the incidence of total maternal complications in the frozen embryo transfer group was significantly higher (30.4% vs. 6.5%, *p*=0.019). Analyzing each complication as a separate entity, patients in the frozen embryo transfer group had a higher incidence of hypertensive disorders of pregnancy (*p*=0.028). Multiple logistic regression analysis showed that frozen embryo transfer was related with an increased occurrence of maternal complications (OR, 6.68, 95% CI, 1.01-44.19, *p*=0.040).

**Conclusions:**

Among patients with endometrial hyperplasia and carcinoma, the rate of live births was comparable between both embryo transfer procedures, while frozen embryo transfer might be associated with a higher risk of maternal complications compared to that with fresh embryo transfer.

## Introduction

Endometrial carcinoma (EC) is a common malignant neoplasm affecting women and its incidence has shown an increasing trend with changes in dietary habits and lifestyles [1]. Endometrial hyperplasia with atypia (AH), also known as endometrial intraepithelial neoplasia (EIN), is a precancerous condition of EC; its rate of progression to cancer is nearly 30-40% [2]. Fertility-sparing treatment has been widely used in patients with EC and AH [3, 4], and previous researches have demonstrated the efficiency progesterone with a high response rate of 75-97% [5, 6]. After complete remission, close follow-up and pregnancy encouragement are recommended because of the high recurrence rate (40-50%), and the median recurrent time is around 12-28 months [6]. However, natural conception is difficult for EC/AH patients because they are often accompanied with causes of infertility, such as obesity and chronic anovulation [7, 8]. Therefore, referral to a reproductive center is highly suggested to expedite treatment with assisted reproduction. *In vitro* fertilization (IVF) has been recognized as an efficient way to increase the probability of pregnancy and birth in EC/AH.

Recently, there has been an increased trend toward implementing frozen embryo transfer (ET) usage in many reproductive centers with the rapid development of embryo freezing techniques [9]. The advantages of frozen ET include reducing the risk of ovarian hyperstimulation syndrome (OHSS) and achieving a physiological endometrial environment for embryo implantation [10, 11]. However, not all infertile women reportedly benefit from the “freeze-all” strategy, and data comparing outcomes of frozen and fresh ET cycles were contradictory. Chen et al. showed that compared with fresh ET, frozen ET achieved a higher live birth rate in patients with polycystic ovary syndrome (PCOS) [12], whereas other researchers did not find significant difference of pregnant outcomes between two ET cycles in ovulatory or non-PCOS women [13, 14]. These inconsistent results suggest that frozen ET is more appropriate in certain groups of patients.

IVF treatment is complicated in EC/AH patients. Frozen ET seems suitable due to the relatively high incidence of OHSS and thin endometrium during ovarian hyperstimulation [15–17]. However, most EC/AH patients are not willing to take more time to wait longer for the frozen ET cycle, considering the high risk of recurrence in the short term. Therefore, doctors may find it difficult to determine whether to continue the fresh ET cycle or cryopreserve the embryos for subsequent frozen ET to achieve a better pregnancy outcome. To date, only four studies have reported IVF characteristics in EC/AH patients, and each study had a small sample size (8, 21, 49, and 75 cycles, respectively) [16–19]. Little is known about the differences between these two ET procedures in this population. The present study aimed to compare outcomes of pregnancies between frozen ET and fresh ET cycles in EC/AH patients.

## Methods

### Study design and population

In this retrospective cohort study, we collected medical records and analyzed data from the Reproductive Center of Peking University Third Hospital (PUTH) between January 2010 and January 2022. Inclusion criteria: (1) histologically-proven endometrial hyperplasia with atypia or well-differentiated endometrioid adenocarcinoma; (2) accepted fertility-sparing treatment and achieved complete remission; (3) underwent standardized controlled ovulation stimulation protocols and achieved embryo transfers; (4) age ≤40 years; and (5) without primary hypertension or diabetes mellitus. Patients with no retrieved oocytes or those with no available embryos to transfer, as well as preimplantation genetic testing (PGT) cycles, were excluded.

**Fig. 1:**
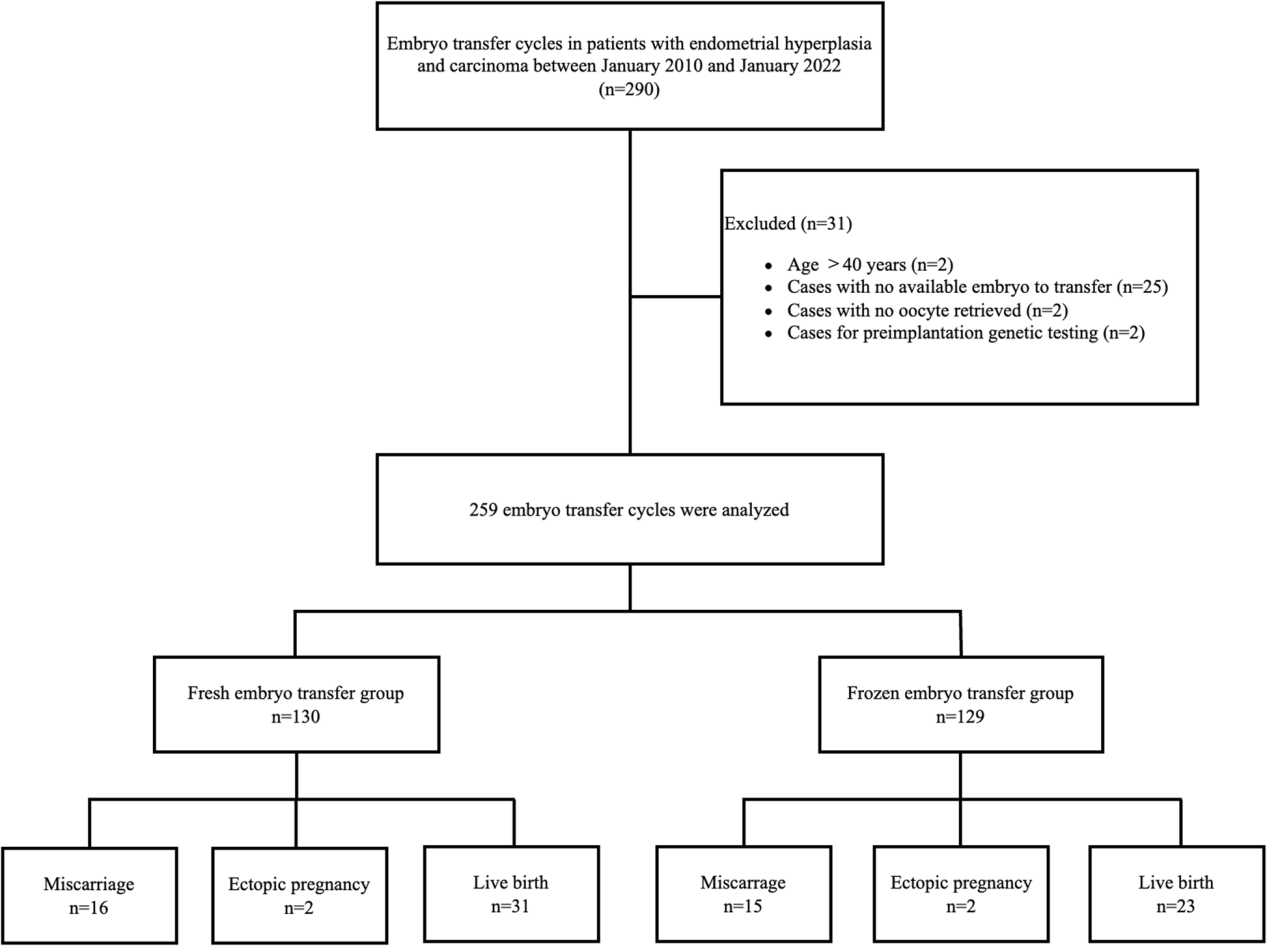
Flow chart of the analysis cohort. AEH endometrial hyperplasia with atypia, EC endometrial cancer, ET embryo transfer.

Figure 1 shows the flow chart of this study. Overall, 290 ET cycles met the screening criteria. After excluding 25 cases with no available embryos to transfer, two cases with no oocytes retrieved, two cases that were aged >40 years, and two cases for the PGT cycle, the remaining 259 cycles were included for analysis and divided into frozen ET and fresh ET groups.

### Ethical consideration

Approval was obtained from the Ethics Committee of the PUTH (No. IRB 00006761-M2020004).

### IVF treatment

The detail of each ovarian stimulation protocol and the process of ovarian follicular monitor have been previously described [20]. Oocyte retrieval was conducted 36 ± 2 hours after triggering. Methods of oocytes fertilization include conventional IVF and intracytoplasmic sperm injection. When embryos were cultivated to day 3, also known as cleavage-stage embryos, they were assessed under microscope. Top-quality cleavage-stage embryos were the embryos that were derived from 2PN embryos, and could reach 5–8 cells with <30% cytoplasmic fragmentation [21]. Non-top-quality cleavage-stage embryos continued to be cultivated to D5 or D6, also known as blastocysts, which were assessed according to the Gardner grading system [22].

Fresh ET was the first choice unless the patient was under special conditions that deemed them not suitable for immediate transfer. Transfer process was performed by a group of experienced doctors. Patients accepted oral or intravaginal progesterone support from the day of oocyte retrieval, and continued to use until 10 weeks of gestation if pregnant.

Some patients did not accept fresh ET owing to the thin endometrium, premature elevation of progesterone, high risk of OHSS, or patient preference. Frozen ET was performed in natural monitored cycles or programmed artificial cycles, which were determined by doctors. Cryopreservation and recovery of viable embryos, regimens for endometrium preparation, and luteal support of frozen ET were conducted according to the protocol at our center [23]. Briefly, the frozen ET was performed in the natural monitored cycle or in the programmed artificial cycle. For the naturally cycle, the thawed embryo was transferred on day 3 or 5 after ovulation. Luteal support was provided with vaginal administration of progesterone 60 mg/d from the night of transfer. For the artificial cycle, the patient took daily oral estradiol to make endometrium development and added oral progesterone when the endometrial thickness was suitable. The thawed embryo was transferred on day 5 after initiation of the progesterone treatment.

During IVF treatment, patients were also followed-up every 3 months by the gynecologic oncologist. Transvaginal ultrasound was performed at each follow-up, and hysteroscopy was performed if irregular vaginal bleeding occurred or abnormality was suggested by ultrasound.

### Study outcomes

The primary outcome was live birth, defined as birth of a live baby beyond 28 weeks of gestation. The secondary study outcomes included perinatal outcomes and other pregnancy outcomes (clinical pregnancy, ectopic pregnancy, miscarriage, and implantation). Perinatal outcomes included maternal complications (hypertensive disorders of pregnancy [HDP], antepartum hemorrhage, gestational diabetes mellitus [GDM]) and neonatal complications (preterm birth, small-for-gestational age [SGA], low birthweight, large-for-gestational age [LGA], and macrosomia).

Serum β human chorionic gonadotropin (β-hCG) levels were measured in all women 14 days after ET. A woman with a positive pregnancy blood test underwent transvaginal ultrasonography at 4 to 6 weeks after ET to identify intrauterine pregnancy, signifying a clinical pregnancy. The implantation rate was calculated as the number of gestational sacs divided by the number of transferred embryos. Ectopic pregnancy was the pregnancy with embryos implanted outside the uterine cavity. Miscarriage was any loss of intrauterine pregnancy before 28 weeks. Ongoing pregnancy was the presence of a fetal heartbeat observed by ultrasonography after 12 weeks. Preterm birth was any delivery event happened between 28 and 37 weeks of gestation. Macrosomia and low birthweight and macrosomia were respectively defined as birth weight ≥4,000 g or <2,500 g. LGA and SGA were defined according to the 10th and 90th percentiles of birth weight reference for the Chinese population [24].

### Statistical analysis

Normally and non-normally distributed continuous variables are respectively presented as mean ± standard deviation and medians (interquartile range [IQR]). And the t-test was used to compare normally distributed continuous variables between two groups, while the Mann–Whitney U-test was used to compare non-normally distributed ones. Categorical variables are presented as numbers (percentages) and were compared using the Pearson’s chi-squared test or Fisher’s exact test. The chi-squared test was not suitable when the total sample size was below forty or the expected values in any of the cells of a contingency table were below 5. Multiple logistic regression analyses were used twice for various purposes. The first multiple analysis aimed to examine the relationship between two ET procedures and pregnancy outcomes, and confounding factors included body mass index, maternal age, histological type, endometrial thickness, infertility type, infertility duration, basal antral follicle count (AFC), basal follicle stimulating hormone (FSH), and the stage of transferred embryos. The second multiple analysis included eight independent variables to identify the factors associated with maternal complication. All analyses were performed using the SPSS software. Significance was defined as a two-sided *p*-value of <0.05.

## Results

### Baseline characteristics

Overall, 259 ET cycles in patients with EC or AH were analyzed, including 129 (49.8%) in the frozen ET group and 130 (50.2%) in the fresh ET group. Table 1, shows the baseline characteristics of this study cohort. Patients in the frozen ET group had more AFC in both ovaries (12.0 vs. 7.0, *p*<0.001) and a higher percentage of PCOS (35.7% vs. 14.6%, *p*<0.001) than patients in the fresh ET group. No significant differences of other baseline characteristics were found between two groups (*p*>0.05). For frozen ET, there were two types of endometrial preparation protocols, including natural monitored cycle (38.8%) and programmed artificial cycle (61.5%).

**Table 1:** Baseline Characteristics of the two embryo transfer procedures

Characteristics	All cycles (*n*=259)	Fresh ET (*n*=130)	Frozen ET (*n*=129)	*p* value
Female age at induction (years)	33.2 ± 3.6	33.3 ± 3.4	33.1 ± 3.8	0.872
Male age at induction (years)	34.3 ± 4.3	34.3 ± 4.2	34.4 ± 4.5	0.818
Body mass index (kg/m^2^)	25.4 ± 3.9	25.9 ± 3.7	25.0 ± 4.1	0.074
Duration of infertility (years)	5.0 (3.0-7.0)	5.0 (3.0-7.0)	5.0 (4.0-7.5)	0.413
Type of infertility				
Primary	188 (72.6)	91 (70.0)	97 (75.2)	0.349
Secondary	71 (27.4)	39 (30.0)	32 (24.8)	
Complications				
Polycystic ovary syndrome	65 (25.1)	19 (14.6)	46 (35.7)	<0.001^*^
Diminished ovarian reserve	51 (19.7)	29 (22.3)	22 (17.1)	0.288
Intrauterine adhesion	12 (4.6)	4 (3.1)	8 (6.2)	0.232
Ovarian reserve				
Basal FSH (IU/L)	5.9 (4.3-7.4)	5.9 (4.3-7.7)	5.8 (4.4-7.2)	0.526
Basal E_2_ (pmol/L)	132.0 (97.6-171.0)	126.5 (96.9-163.3)	142.0 (104.5-187.0)	0.069
No. of basal AFC	8.0 (5.0-16.0)	7.0 (4.8-11.3)	12.0 (6.0-18.0)	<0.001^*^
Histological type				
AH	189 (73.0)	97 (74.6)	92 (71.3)	0.550
EC	70 (27.0)	33 (25.4)	37 (28.7)	
Treatment duration (months)	6.0 (4.0-9.0)	6.0 (4.0-9.0)	6.0 (5.0-9.0)	0.745
No. of hysteroscope	3.0 (2.0-4.0)	3.0 (2.0-4.0)	3.0 (2.0-4.0)	0.714
Endometrial thickness on ET days	9.0 (8.0-11.0)	10.0 (8.0-11.0)	9.0 (8.0-10.0)	0.290
≤7 mm	40 (15.4)	17 (13.1)	23 (17.8)	
>7 mm	219 (84.6)	113 (86.9)	106 (82.2)	
Mode of delivery				
Vaginal delivery	17 (31.5)	7 (22.6)	10 (43.5)	0.102
Caesarean section	37 (68.5)	24 (77.4)	13 (56.5)	
Endometrial preparation for frozen ET				
Natural monitored cycle			50 (38.8)	
Programmed artificial cycle			79 (61.5)	

Data are presented as n (%), median (interquartile range), or mean ± standard deviation.

*AFC* Antral follicle count, *AH* Endometrial hyperplasia with atypia, *EC* Endometrial carcinoma, ET eEmbryo transfer.

^*^p <0.05

### Live birth rate and other pregnancy outcomes

Pregnancy outcomes are listed in Table 2. A total of 59 live births, including 49 singletons and five twins, were achieved. Overall, 54 out of 259 cycles achieved live birth and the whole live birth rate per ET cycle was 20.8%, which was 17.8% (23/129) in the frozen ET group and 23.8% (31/130) in the fresh ET group. No significant between-group difference was observed after adjusting for potential confounding factors (adjusted OR, 0.47; 95% CI, 0.21-1.06; *p*=0.068). Both groups had comparable implantation rate, clinical pregnancy rate and ongoing pregnancy rate. (*p*=0.411, 0.258 and 0.248, respectively).

**Table 2:** Pregnancy outcomes of the two embryo transfer procedures

Outcome	Fresh ET (*n*=130)	Frozen ET (*n*=129)	Unadjusted			Adjusted	
			COR (95%CI)	*p* value		aOR (95%CI)	*p* value
Live birth rate	31(23.8)	23(17.8)	0.69(0.38-1.27)	0.235		0.47(0.21-1.06)	0.068
Singleton	27 (87.1)	22 (95.7)					
Twins	4 (12.9)	1 (4.3)					
Implantation (per embryo)	56/229 (24.5)	42/199 (21.1)		0.411			
Clinical pregnancy	49 (37.7)	40 (31.0)	0.74(0.44-1.24)	0.258		0.68(0.35-1.29)	0.233
Ongoing pregnancy among clinical pregnancy	33 (25.4)	25 (19.4)	0.71(0.39-1.27)	0.248		0.46(0.21-1.03)	0.058
Ectopic pregnancy among clinical pregnancy	2 (4.1)	2 (5)	1.01(0.14-7.27)	0.994		1.39(0.10-19.88)	0.806
Miscarriage among clinical pregnancy	16 (32.7)	15 (37.5)	0.94(0.46-1.93)	0.875		1.19(0.50-2.81)	0.691
First trimester	14 (87.5)	13 (86.7)					
Second trimester	2 (12.5)	2 (13.3)					

Data are presented as n (%).

*COR* Crude odds ratio, *CI* Confidence interval, Aor Adjusted odds ratio.

Of 259 ET cycles, 89 (34.4%) clinical pregnancies were recorded. The miscarriage rate per clinical pregnancy was 34.8% (31/89), of which early and late abortion rates were 20.3% (27/89) and 4.5% (4/89), respectively. Among the four patients who experienced miscarriages at 12-28 weeks of gestation, twin pregnancies of two patients were inevitably aborted because of preterm premature rupture of membranes, one patient experienced spontaneous abortion because of suspected cervical incompetence, and one patient underwent induced abortion owing to intrauterine fetal death for unknown reasons.

### Neonatal complications

As shown in Table 3, the mean gestational age was 37.7 ± 2.8 and 38.3 ± 1.5 weeks in the frozen ET and fresh ET groups, respectively (*p*=0.653). The incidence of preterm birth among all deliveries was similar between two groups (16.1% vs. 17.4%, *p*=0.902). The mean birth weights of singletons in two groups were 3429.1 ± 583.7 g and 3259.3 ± 371.8 g, respectively (*p*=0.472). There were no significant between-group differences in terms of incidence of SGA, LGA, low birthweight, and macrosomia among all live newborns (*p*=0.264, 0.294, 0.294 and 0.294, respectively).

**Table 3:** Maternal and perinatal complications of two embryo transfer procedures

Outcome	All cycles	Fresh ET	Frozen ET	*p* value
**Maternal complications** no./total no. of deliveries (%)				
Total	9 (16.7)	2 (6.5)	7 (30.4)	0.019^*^
Gestational diabetes mellitus	4 (7.4)	2 (6.5)	2 (8.7)	>0.999^#^
Hypertensive disorders of pregnancy	4 (7.4)	0	4 (17.4)	0.028^#*^
Antepartum haemorrhage	1 (1.9)	0	1 (4.3)	0.426^#^
**Perinatal outcomes** no./total no. (%)				
Gestational age at birth (weeks)	38.0 ± 2.1	38.3 ± 1.5	37.7 ± 2.8	0.653
Birth weight (g)				
Singleton	3335.5 ± 480.9	3259.3 ± 371.8	3429.1 ± 583.7	0.472
Twin	2611.0 ± 397.5	2720.0 ± 365.6	2175.0 ± 106.1	0.185
Sex of infants				
Female	34/59 (57.6)	21/35 (60.0)	13/24 (54.2)	0.656
Male	25/59 (42.4)	14/35 (40.0)	11/24 (45.8)	
Preterm birth among all deliveries	9/54 (16.7)	5/31 (16.1)	4/23 (17.4)	0.902
Singleton	6/49 (12.2)	3/27 (11.1)	3/22 (13.6)	
Twin	3/5 (60.0)	2/4 (50.0)	1/1 (100.0)	
Low birthweight among all live newborns	4/59 (6.8)	1/35 (2.9)	3/24 (12.5)	0.294^#^
Macrosomia among all live newborns	4/59 (6.8)	1/35 (2.9)	3/24 (12.5)	0.294^#^
SGA among all live newborns	3/59 (5.1)	3/35 (8.6)	0	0.264^#^
LGA among all live newborns	4/59 (6.8)	1/35 (2.9)	3/24 (12.5)	0.294^#^

Data are presented as n (%) or mean ± standard deviation.

*LGA* Large-for-gestational age, *SGA* Small-for-gestational age.

^#^Fhisher’ exact test.

^*^p <0.05.

### Maternal complications

Nine (16.7%) maternal complications were recorded, of which four were HDP, four were GDM and one was antepartum hemorrhage. The incidence of total maternal complications was significantly higher in the frozen ET group (30.4% vs. 6.5%, *p*=0.019). When analyzing each complication as a separate entity, compared with patients in the fresh ET group, patients in the frozen ET group had a higher prevalence of HDP (0 vs. 17.4%, *p*=0.028), whereas no significant differences were identified in the incidences of antepartum hemorrhage (0 vs. 4.3%, *p*=0.426) and GDM (6.5% vs. 8.7%, *p*>0.999).

Logistic regression analysis was performed to find factors might be related with overall maternal complications (Table 4). We included eight independent variables for multiple analyses, and the results showed that frozen ET was associated with an increased occurrence of maternal complications (OR, 6.68, 95% CI, 1.01-44.19, *p*=0.040).

**Table 4:** Univariate and multiple logistic regression analysis in maternal complications

Variables	Univariate analysis			Multiple analysis	
	OR (95%CI)	*p* value		OR (95%CI)	*p* value
Age (years)		0.378			0.512
<35	1			1	
≥35	0.51 (1.12–2.25)			0.53 (0.08-3.55)	
Histological type		0.453			0.535
AEH	1			1	
EC	0.43 (0.05–3.87)			0.43 (0.03-6.25)	
BMI (kg/m^2^)		0.977			0.606
<24	1			1	
≥24	0.98 (0.22–4.38)			1.63 (0.26-10.39)	
Duration of infertility (months)		0.754			0.683
<5	1			1	
≥5	1.25 (0.31–5.06)			0.66 (0.09-4.88)	
Type of infertility		0.114			0.247
Primary	1			1	
Secondary	1.18 (0.02-1.52)			0.23 (0.02-2.79)	
FSH (mIU/mL)	1.05 (0.82-1.33)	0.716		1.06 (0.72-1.55)	0.786
Complication of PCOS	3.40 (0.82-14.15)	0.093		1.85 (0.27-12.67)	0.532
Transfer procedure		0.016^*^			0.040^*^
Fresh ET	1			1	
Frozen ET	7.73 (1.46-41.09)			6.68 (1.01-44.19)	

*AH* Endometrial hyperplasia with atypia, *EC* Endometrial carcinoma, *PCOS* Polycystic ovarian syndrome, *ET* Embryo transfer, *OR* Odd ratio, *CI* Confidence internal.

^*^p <0.05.

## Discussion

This retrospective study showed IVF-related pregnancy outcomes in a large sample of EC/AH patients. To the best of our knowledge, this is the first study to compare perinatal outcomes after frozen and fresh ET in this population. We found that live birth rates were comparable between two ET procedures, although an increasing trend was observed after fresh ET cycles. We also found significant increase in the incidence of maternal complications and pregnancy-induced hypertension in the frozen ET cycles. Our finding is consistent with previous studies, that is there is a comparable live birth rate between two ET procedures in normo-responder patients [25, 26]. The exact reason for the increased rate of hypertension in frozen ET is still unknown, which might be due to the non-physiological concentrations of exogenous progesterone and estrogen in artificial cycles [27].

Having a healthy baby is the ultimate goal of EC/AH patients receiving fertility-sparing treatment, and IVF-ET has been suggested as the most efficient way to achieve pregnancy. Most previous studies have only reported the total number of patients who finally conceived both naturally or through IVF, and the cumulative live birth rate of EC/AEH patients ranged from 40% to 80% [6, 16, 17, 28]. In clinical practice, patients are also concerned about the probability of live birth after each ET cycle. To date, only two studies have reported the rate of live birth per ET cycle, which was 14.3% and 17.3%, respectively [18, 19]. In our study, 54 out of 289 cycles achieved live birth and the live birth rate was 20.8%, which was consistent with that seen in previous studies[18, 19]. However, to the best of our knowledge, no studies have compared outcomes following frozen and fresh ET cycles in EC/AH patients.

There is ongoing debate regarding which ET procedure can achieve a higher rate of live births. In a randomised controlled trial (RCT), Chen and colleagues reported frozen ET resulted in a higher live birth rate per first transfer cycle compared to that with fresh ET in 1508 PCOS patients [12]. Another large multicenter RCT also demonstrated an increased singleton live birth rate following frozen single blastocyst transfer in women with normal ovulation [29], whereas most other RCTs showed no difference [13, 14]. Two previous meta-analyses, including different RCTs, suggested that frozen ET is associated with higher rates of live births following the first transfer [25, 26]. Subgroup analysis indicated that this significant difference only occurred in PCOS/hyper-responder patients, whereas there was no difference in non-PCOS/normo-responders, suggesting that frozen ET is more appropriate for specific populations which are PCOS/hyper-responders. These patients usually have a high risk of OHSS after oocyte retrieval, which may damage embryo implantation and increase the abortion rate in fresh ET cycles [12, 29]. Therefore, these patients may achieve a higher live birth rate in frozen ET cycles. Considering our study population, in this retrospective study, our results showed similar live birth and clinical pregnancy rates between two ET procedures. These findings indicate that a “freeze-all” strategy may not be appropriate for all EC/AH patients, and that frozen ET is recommended only when specific indications exist.

Several cohort studies and meta-analyses have indicated an increased risk of pregnancy-induced hypertension following frozen ET compared with that in fresh ET. Opdahl et al. analyzed pregnancies in the same women and found that frozen ET was associated with a higher risk of HDP compared to that with fresh ET (OR, 2.63; 95% CI, 1.73-3.99) [30]. A recent Cochrane review including 3940 women from three RCTs also demonstrated the increased risk of hypertensive disorders in “freeze all” strategy (OR, 2.15; 95% CI, 1.42-3.25) [31]. Our findings also support the existing literature, and we found that the prevalence of HDP was higher with frozen ET than that with fresh ET. Additionally, further studies have explored the relationship between perinatal outcomes and different regimens of endometrial preparation in frozen ET; the results indicated an increased rate of hypertensive disorders in artificial cycles [32]. One of the proposed causes for these differences is the effect of non-physiological concentrations of exogenous progesterone and estrogen during artificial cycles [27]. Another possible reason is the lack of corpus luteum function in artificial cycles [33].

Meta-analyses of observational studies and RCTs have demonstrated an increased risk of LGA and a lower rate of SGA in singletons born as a result of fresh ET [25, 34]. The outcomes of sibling pregnancies indicated that frozen ET was associated with a higher live birthweight [35, 36]. In our study, although there tends a higher rate of LGA in frozen ET group, the difference was not statistically significant (12.5% vs. 2.9%, p=0.294). However, the underlying mechanism is still unclear. One explanation is that the endometrium in the natural cycles of frozen ET is not influenced by supraphysiological serum estradiol concentrations [37]. It has been also assumed that epigenetic event happens during the process of embryo freezing and thawing, which may induce changes of fetal growth potential [38]. It must be highlighted, however, that confounders, such as gestational weight gain, the presence of gestational diabetes, and pre-pregnancy body mass index were not assessed in previous studies. Further studies with larger samples are necessary to obtain confirmed results and investigate the underlying mechanisms.

PGT includes a series of genetic assays used to evaluate embryos prior to transfer to the uterus. After embryos are retrieved and fertilized, the embryologist will perform assisted hatching on embryos at the blastocyst stage to help obtain several cells from the trophectoderm layer, and these cells will be sent for genetic analysis. The current indication for PGT includes couples with monogenic disease or structural chromosomal abnormalities, women with advanced age, recurrent pregnancy loss or repeated implantation failure, and male factor infertility. Therefore, most patients who applied for PGT have a high risk of implantation failure or abortion, and most embryos transferred in PGT cycles have been tested to rule out the possibility of aneuploidy. Considering these confounding factors, we exclude PGT cycles for analysis in this study.

Our study has some limitations. First, selection bias may have occurred owing to the retrospective nature of this study. We can see from Table 1 that the number of basal AFC and the percentage of PCOS were significantly different between two groups. Therefore, to achieve the consolidated results, we performed multiple logistic regression analysis to control for these covariates. Second, although the sample size in this study was relatively large, the interpretation of maternal and neonatal outcomes was limited by the data available for analysis. Therefore, the generalizability of our findings is limited by the sample size and the single center setting. It will be necessary to conduct randomized trials with large samples to further confirm this issue. Finally, some factors that might be related with perinatal outcomes, including the number and quality of transferred embryos, ovarian stimulation regimens, and the total dose of gonadotropins, were not analyzed in this study.

## Conclusions

In conclusion, our study showed that in EC/AH patients who underwent IVF treatment, the live birth rate was comparable between the two ET procedures, while frozen ET might be associated with a higher incidence of maternal complications compared to that with fresh ET. Our findings suggest that patients with EC/AH may not benefit from a “freeze-all” management, and that frozen ET is recommended under specific circumstances. In the future, more well-designed RCTs are required to further elucidate this issue.

## Data Availability

All data generated or analyzed during this study are included in this published article.

## Abbreviations

AH: Endometrial hyperplasia with atypia
AFC: Antral follicle count
EC: Endometrial cancer
FSH: Follicle stimulating hormone
GDM: Gestational diabetes mellitus
HDP: Hypertensive disorders of pregnancy
IVF-ET: *In vitro* fertilization and embryo transfer
LGA: arge-for-gestational age
OHSS: Ovarian hyperstimulation syndrome
PCOS: Polycystic ovary syndrome
PUTH: Peking University Third Hospital
RCT: randomised controlled trial
Hcg: Human chorionic gonadotropin
SGA: Small-for-gestational age
